# Breast cancer cell cyclooxygenase-2 expression alters extracellular matrix structure and function and numbers of cancer associated fibroblasts

**DOI:** 10.18632/oncotarget.14912

**Published:** 2017-01-31

**Authors:** Balaji Krishnamachary, Ioannis Stasinopoulos, Samata Kakkad, Marie-France Penet, Desmond Jacob, Flonne Wildes, Yelena Mironchik, Arvind P. Pathak, Meiyappan Solaiyappan, Zaver M. Bhujwalla

**Affiliations:** ^1^ JHU ICMIC Program, Division of Cancer Imaging Research, The Russell H. Morgan Department of Radiology and Radiological Science, Baltimore, MD 21205, USA; ^2^ Sidney Kimmel Comprehensive Cancer Center, The Johns Hopkins University School of Medicine, Baltimore, MD 21205, USA

**Keywords:** COX-2, cancer associated fibroblasts, collagen 1 fibers, macromolecular transport, metastasis

## Abstract

Cyclooxygenase-2 (COX-2) is a critically important mediator of inflammation that significantly influences tumor angiogenesis, invasion, and metastasis. We investigated the role of COX-2 expressed by triple negative breast cancer cells in altering the structure and function of the extracellular matrix (ECM). COX-2 downregulation effects on ECM structure and function were investigated using magnetic resonance imaging (MRI) and second harmonic generation (SHG) microscopy of tumors derived from triple negative MDA-MB-231 breast cancer cells, and a derived clone stably expressing a short hairpin (shRNA) molecule downregulating COX-2. MRI of albumin-GdDTPA was used to characterize macromolecular fluid transport *in vivo* and SHG microscopy was used to quantify collagen 1 (Col1) fiber morphology. COX-2 downregulation decreased Col1 fiber density and altered macromolecular fluid transport. Immunohistochemistry identified significantly fewer activated cancer associated fibroblasts (CAFs) in low COX-2 expressing tumors. Metastatic lung nodules established by COX-2 downregulated cells were infrequent, smaller, and contained fewer Col1 fibers.

COX-2 overexpression studies were performed with tumors derived from triple negative SUM-149 breast cancer cells lentivirally transduced to overexpress COX-2. SHG microscopy identified significantly higher Col1 fiber density in COX-2 overexpressing tumors with an increase of CAFs. These data expand upon the roles of COX-2 in shaping the structure and function of the ECM in primary and metastatic tumors, and identify the potential role of COX-2 in modifying the number of CAFs in tumors that may have contributed to the altered ECM.

## INTRODUCTION

Cyclooxygenase-2 (COX-2) is an active mediator of the inflammatory response of cells [1]. Its major role in a multitude of degenerative diseases such as autoimmune diseases [2], gastric inflammation [3], and several cancers, such as gastric [4], lung [5], breast [6, 7] and colon cancer [8], has resulted in the development of pharmaceutical inhibitors targeting COX-2. However the side effects of these agents [9, 10] have diminished the prospects of their use in cancer treatment. Nevertheless, COX-2 remains one of the most important targets in cancer, especially for cancers that are COX-2-dependent [7, 11–13]. The promise of molecular agents such as small interfering RNA (siRNA), that are more specific than pharmacological interventions, provides renewed hope for exploiting this target [14].

In breast cancer, several studies have highlighted the importance of COX-2 in tumor development, progression, invasion, and metastasis [15–17]. Uncovering mechanisms by which COX-2 regulates these processes can provide new insights and identify novel targets. In a study of 127 patients, triple negative breast cancer (TNBC) was found to be an independent predictor for COX-2 overexpression [18]. Silencing COX-2 in MDA-MB-231 metastatic TNBC cells inhibited tumor onset and growth in an orthotopic xenograft model, and inhibited pulmonary colonization in an experimental model of metastasis [17]. These changes were attributed to reduced invasiveness, reduced angiogenic capabilities, and reduced expression of pro-metastatic components of the extracellular matrix (ECM) [19]. COX-2 inhibition has been found to significantly reduce the expression of degradative enzymes such as matrix metalloproteinase 1 (MMP1), and alter the expression of ECM components such as hyaluronan and lumican that play a role in intra-fibrillar collagen spacing [17, 19]. The role of prostaglandins produced by COX-2 in promoting cancer cell adhesion in the ECM has been extensively reviewed [20].

More recently, COX-2 has been investigated within the context of ECM modification. Pharmacological inhibition of COX-2 was found to reduce collagen deposition and tumor growth in the MMTV-PyMT or MMTV-PyMT/Col1a1 mouse models [21], and invasion during mammary gland involution [21, 22]. The alignment of collagen fibers perpendicularly to the tumor boundary was also associated with decreased disease-free survival in breast cancer patients [22]. The role of COX-2 mediated collagen deposition and remodeling in breast cancer metastasis is also being actively investigated [21, 23]. A high density of collagen 1 (Col1) fibers in the tumor ECM has been identified as a predictor of increased metastasis [24, 25].

Here we investigated the role of COX-2 expression by TNBC cells in shaping the structure and function of the tumor ECM. Studies were performed in triple negative MDA-MB-231 tumors derived from cells with COX-2 downregulated by stable expression of COX-2 short hairpin RNA (shRNA) and in triple negative SUM-149 tumors derived from cells with COX-2 overexpressed following lentiviral transduction. These tumors were used to investigate the relationship between COX-2 expression, vascular parameters, and macromolecular transport, using MRI, and Col1 fiber distribution, using second harmonic generation (SHG) confocal microscopy. We investigated the ability of these cells to spontaneously metastasize to the lymph nodes and to establish metastatic nodules in lungs in an experimental model of metastasis. Col1 fiber patterns in the lung nodules were characterized. While cancer cells shape Col1 fiber patterns through the secretion of various enzymes [26], Col1 fiber is primarily synthesized by activated cancer associated fibroblasts (CAFs) within the tumor [27]. We therefore quantified the number of activated CAFs in the tumors using immunohistochemistry and immunoblotting for alpha-smooth muscle actin (α-SMA).

We identified significant differences in vascular permeability and macromolecular transport in COX-2 downregulated MDA-MB-231 tumors together with a significant decrease of vascular endothelial growth factor (VEGF) that explained the decrease of vascular permeability detected with MRI. Sparser Col1 fibers were evident with COX-2 downregulation in primary tumors together with fewer and smaller metastatic nodules. Both primary tumors and metastatic nodules contained fewer CAFs.

COX-2 overexpressing SUM-149 tumors displayed increased Col1 fiber density with a higher number of CAFs. These data expand upon the role of COX-2 in modifying the structure and function of the ECM, and identify the potential role of COX-2 in activating fibroblasts in the tumor.

## RESULTS

**Figure 1:**
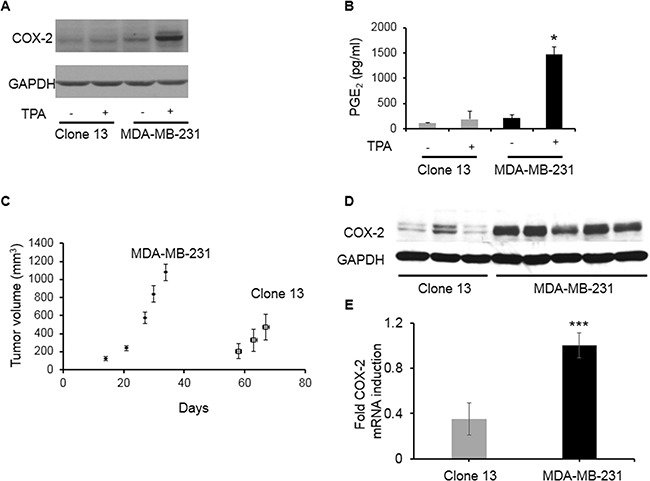
**A**. COX-2 expression in cells. **B**. PGE_2_ expression in cells; cells were exposed to 50 nM TPA for 24 h to induce COX-2 expression. **C**. Tumor volumes for COX-2 containing parental MDA-MB-231 (N=5) and COX-2 reduced Clone 13 (N=8) tumors; 2 × 10^6^ cells were inoculated in 0.1 ml of 8.8 mg/ml Matrigel. **D**. Representative immunoblot showing COX-2 expression in MDA-MB-231 and Clone 13 tumors. GAPDH was used as loading control. **E**. Relative fold change in COX-2 mRNA levels in MDA-MB-231 (N=6) and Clone 13 (N=4) tumors. Values represent Mean ± SEM. *p ≤ 0.05; ***p ≤ 0.001 using ΔC_t_ values.

COX-2 levels were significantly lower in MDA-MB-231 Clone 13 cells; these cells could only be moderately induced to express COX-2 with 12-O-tetradecanoylphorbol-13-acetate (TPA) (Figure 1A), and secrete the COX-2 product prostaglandin E_2_ (PGE_2_) following induction with TPA (Figure 1B). Injection of COX-2-reduced Clone 13 cells with Matrigel in the mammary fat pad gave rise to tumors with significantly delayed onset as shown in growth curves (Figure 1C) that correlated well with shRNA-mediated reduction of COX-2 and COX-2-catalyzed PGE_2_ formation. The decrease in COX-2 expression in tumors derived from Clone 13 cells were confirmed from protein expression (Figure 1D) and mRNA levels (Figure 1E).

**Figure 2:**
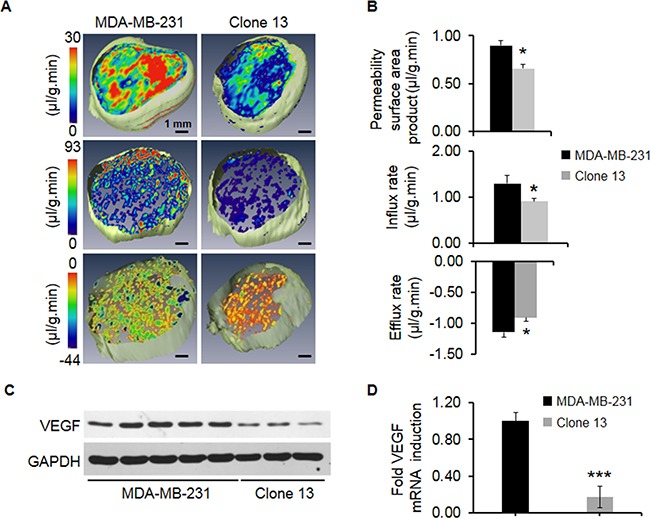
**A**. Representative 3D maps of permeability surface area product (top), influx rate (middle), and efflux rate (bottom) for high COX-2 expressing parental MDA-MB-231 and COX-2 reduced Clone 13 tumors. **B**. Quantitative comparisons of permeability surface area product (top), influx rate (middle) and efflux rate (bottom) in high COX-2 expressing parental MDA-MB-231 (N=6) and COX-2 reduced Clone 13 (N=6) tumors. Significantly lower permeability (p-value = 0.003), influx rates (p-value =0.045) and efflux rates (p-value = 0.036) were observed in COX-2 reduced Clone 13 tumors as compared to COX-2 containing parental MDA-MB-231 tumors. **C**. Representative immunoblot showing VEGF expression in MDA-MB-231 and Clone 13 tumors. GAPDH was used as a loading control. **D**. Relative fold change of VEGF mRNA expression in MDA-MB-231 (N=6) and Clone 13 (N=4) tumors. Values represent Mean ± SEM. ***p ≤ 0.001 using ΔC_t_ values

To evaluate functional changes in the ECM we injected the macromolecular contrast agent albumin-GdDTPA (~100 kDa) *i.v*. and followed its *in vivo* distribution noninvasively in volume-matched tumors. This allowed us to derive macromolecular transport parameters as well as evaluate the permeability of the tumor vasculature to this contrast agent. Representative MR derived images of permeability (Figure 2A, top), influx rate (Figure 2A, middle) and efflux rate (Figure 2A, bottom) show the effect of COX-2 reduction on permeability and macromolecular transport. Quantification of these parameters is shown in Figure 2B for permeability (top), influx rate (middle) and efflux rate (bottom). Permeability and macromolecular transport were significantly lower in COX-2 downregulated Clone 13 tumors. The scale in the efflux rate panel is inverted with cooler colors reflecting faster draining of the contrast agent. A significant decrease of VEGF protein (Figure 2C) and mRNA (Figure 2D) was observed in Clone 13 tumors.

**Figure 3:**
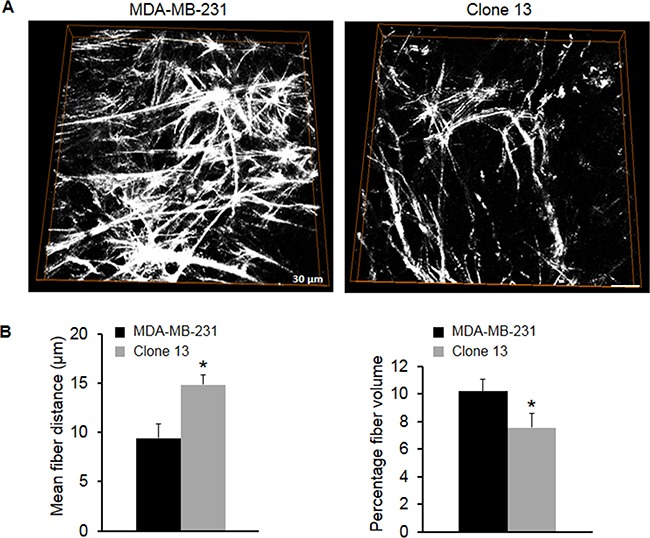
**A**. 3D visualization of Col1 fibers in COX-2 containing parental MDA-MB-231 and COX-2 reduced Clone 13 tumors. The FOV image size was 334.91 × 334.91 × 15 μm^3^ with a voxel size of 0.66 × 0.66 × 1 μm^3^. **B**. Quantification of Col1 fiber volume and fiber distribution. COX-2 reduced Clone 13 tumors (N=7) had significantly larger inter-fiber distance and significantly lower percent fiber volume compared to COX-2 containing parental MDA-MB-231 tumors (N=5). Values represent Mean ± SEM. *p ≤ 0.05.

To evaluate the effect of COX-2 expression on structural ECM changes, we characterized Col1 fiber distribution in 1 mm-thick fresh tumor slices using second harmonic generation (SHG) microscopy. Representative images of Col1 fibers from a z-stack are displayed in Figure 3A that demonstrate the reduced Col1 fiber content in Clone 13 tumors compared to MDA-MB-231 tumors. Clone 13 tumors with COX-2 downregulated contained fewer Col1 fibers with significantly increased mean inter-fiber distance (Figure 3B, left) and reduced fractional fiber volume (Figure 3B, right).

**Figure 4:**
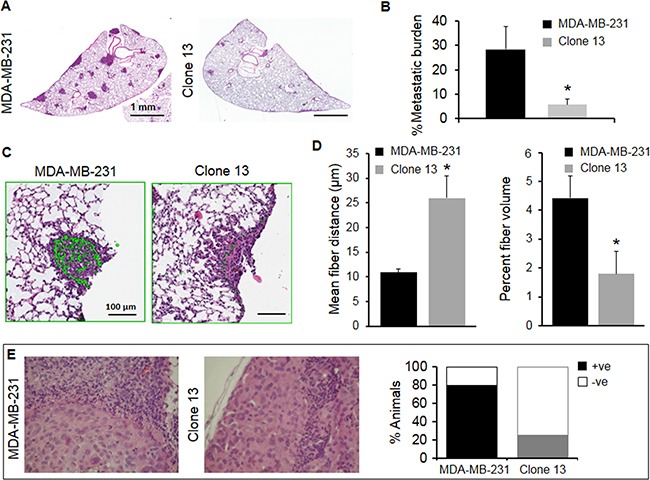
**A**. Representative examples of H&E stained tumor sections of lungs obtained from mice intravenously injected with 10^6^ MDA-MB-231 or Clone 13 cells. **B**. Metastatic burden was calculated as [(Total area of metastatic foci in μm^2^)/(Total lung area in μm^2^)]x100. Metastatic burden from MDA-MB-231 injected mice (N=5) was significantly higher (p=0.059) compared to metastatic burden from Clone 13 injected mice (N=3). Values represent Mean ± SEM. *p ≤ 0.06. **C**. Representative images of Col1 fiber distribution in metastatic lung nodules obtained with SHG microscopy overlaid on the corresponding H&E stained region, from mice intravenously injected with 10^6^ MDA-MB-231 or Clone 13 cells. **D**. Quantification of Col1 fiber volume and fiber distribution in lung nodules. Lung nodules obtained from mice injected with COX-2 reduced Clone 13 (N=3) cells had significantly larger inter-fiber distance (p-value = 0.053) and significantly lower percent fiber volume (p-value = 0.049) compared to COX-2 containing parental MDA-MB-231 mice (N=5). Values represent Mean ± SEM. *p ≤ 0.05. **E**. Representative photomicrographs of H&E stained sections of lymph nodes with cancer cells. Four of five MDA-MB-231 tumor-bearing mice had cancer cells detected in the axillary lymph nodes and one of four Clone 13 tumor-bearing mice had cancer cells detected in the axillary lymph nodes.

COX-2 downregulation in MDA-MB-231 cells resulted in fewer and smaller metastatic lung nodules in an experimental model of metastasis. Representative hematoxylin and eosin (H&E) stained lung sections, shown in Figure 4A, demonstrate the reduction in colonization and establishment of pulmonary metastasis following COX-2 downregulation. Figure 4B shows the significant decrease of metastatic burden observed following COX-2 downregulation.

Metastatic lung nodules established by Clone 13 cells had fewer Col1 fibers in the nodules compared to nodules established by COX-2 expressing MDA-MB-231 cells (Figure 4C). Quantification of inter-fiber distance and fiber volume shown in Figure 4D revealed a significant difference of both parameters in lung nodules following COX-2 reduction. To establish a relationship between COX-2 downregulation in primary tumors and lymph node metastasis, H&E stained axillary lymph node sections were analyzed for presence of cancer cells. As shown in Figure 4E, 80% of animals were positive for presence of cancer cells in axillary lymph nodes in the MDA-MB-231 tumor group compared to 20% in the Clone 13 tumor group.

**Figure 5:**
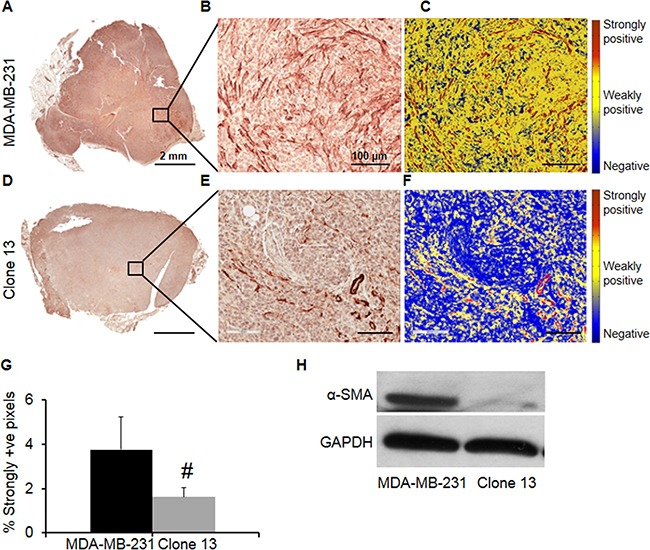
Representative images of α-SMA immunostained sections obtained from **A**. MDA-MB-231 and **D**. Clone 13 tumors. Magnified FOVs showing immunostained CAFs and the accuracy of the algorithm in identifying CAFs are presented in **B**. and **C**. for the MDA-MB-231 tumor section and in **E**. and **F**. for the Clone 13 tumor section. **G**. Quantification of immunostaining identified the presence of higher CAFs in MDA-MB-231 tumors (N=5) compared to Clone 13 tumors (N=6). Values represent Mean ± SEM. #p ≤ 0.084. **H**. Representative α-SMA immunoblot obtained from an MDA-MB-231 and a Clone 13 tumor. GAPDH was used as a loading control.

COX-2 downregulation decreased the presence of CAFs in primary tumors. Representative images of α-SMA immunostained sections obtained from MDA-MB-231 and Clone 13 tumors are shown in Figures 5A and 5D respectively. Magnified FOVs showing immunostained CAFs and the image segmentation used to identify the fibroblasts are presented in Figures 5B and 5C for the MDA-MB-231 tumor section, and in Figures 5E and 5F for the Clone 13 tumor section. Since smooth muscle cells also express α-SMA [28], vessel regions were excluded in the analysis. Quantification of immunostaining identified higher CAFs in MDA-MB-231 tumors compared to Clone 13 tumors, as shown in Figure 5G. Representative α-SMA immunoblots obtained from an MDA-MB-231 and a Clone 13 tumor are presented in Figure 5H and demonstrate the decrease of α-SMA expression following COX-2 downregulation.

**Figure 6:**
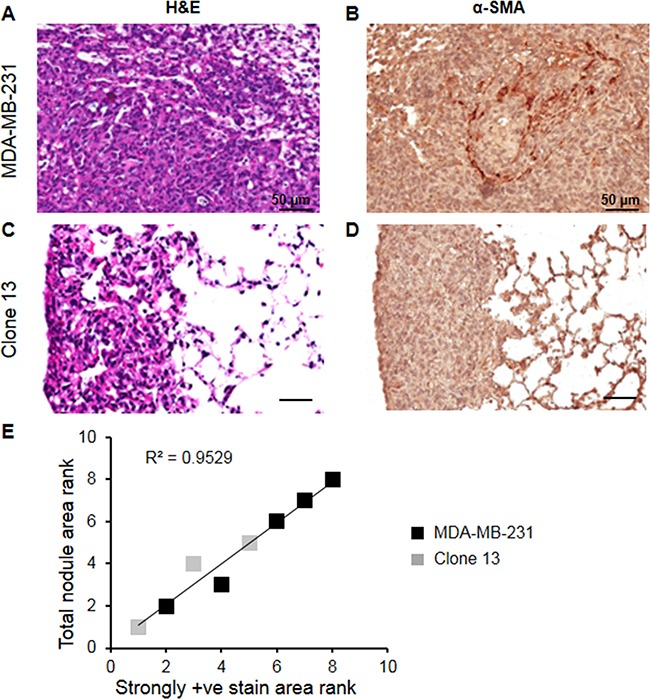
Representative 5 μm-thick H&E and corresponding α-SMA immunostained sections obtained from lungs of mice injected with **A, B**. MDA-MB-231 (N=5) and **C, D**. Clone 13 (N=3) cells. **E**. Spearman correlation between sum of metastatic nodule pixels (reflecting total nodule area) and sum of strongly positive pixels (reflecting number of CAFs) in lungs obtained from each mouse. A significant correlation was observed supporting the role of CAFs in the formation of metastasis.

As shown in representative 5 μm-thick H&E and corresponding α-SMA immunostained sections obtained from lungs of mice injected with MDA-MB-231 (Figures 6A and 6B) and Clone 13 (Figures 6C and 6D) cells, fewer CAFs were observed in Clone 13 lung nodules that were also typically smaller. Lung nodules obtained from mice injected with MDA-MB-231 or Clone 13 cells in the tail vein revealed a significant correlation between nodule size and the number of CAFs. A significant correlation was observed between the sum of metastatic nodule pixels (reflecting total nodule area) and the sum of strongly positive pixels (reflecting number of CAFs) in lungs obtained from each mouse (Figure 6E), supporting the role of CAFs in the formation of metastasis.

**Figure 7:**
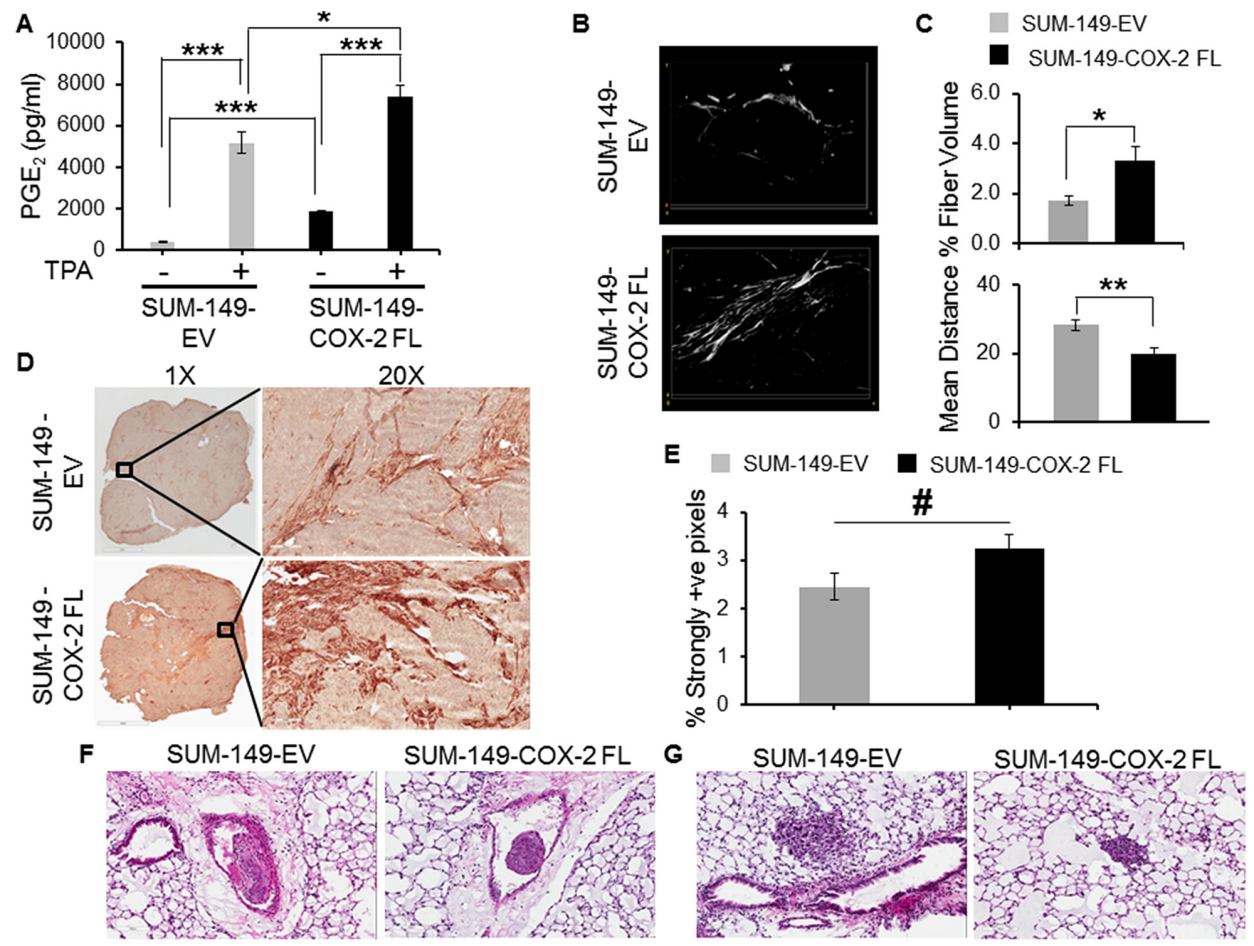
**A**. PGE_2_ expression in COX-2 overexpressing cells; cells were exposed to 50nM TPA for 24hrs to induce COX-2 expression. Values represents Mean ± SEM from four independent experiments; * p≤ 0.005. **B**. 3D visualization of Col1 fibers in empty vector expressing and COX-2 overexpressing SUM-149 tumors. The FOV image size was 423.5×423.5×12μm^3^. **C**. Quantification of Col1 fiber volume and fiber density. COX-2 overexpressing tumors (N=5) had significantly lower inter-fiber distance and significantly higher percent fiber volume compared to SUM-149-EV tumors (N=5). Values represent Mean ± SEM. * p≤ 0.05, ** p≤ 0.005. **D**. Representative images of α-SMA immunostained sections obtained from SUM-149-EV (top) and SUM-149-COX-2FL (bottom) tumors. Magnified FOVs at 20X show immunostained CAFs for SUM-149-EV (top) and SUM-149-COX-2FL (bottom) tumors. **E**. Quantification of immunostained sections identified a higher number of CAFs in SUM-149-COX-2FL tumors (N=5) compared to SUM-149-EV tumors (N=5). Values represent Mean ± SEM. # p=0.06. **F**. Representative high resolution 20X H&E images of lung section showing emboli formation following intravenous injection of SUM-149-EV cells (left) and SUM-149-COX-2FL cells (right). **G**. Representative high-resolution 20X images of lung showing pulmonary metastatic foci following intravenous injection of SUM-149-EV cells (left) and SUM-149-COX-2FL (right).

To further establish the role of COX-2 expression in modulating the ECM, we stably overexpressed the coding sequence of COX-2 in SUM-149 breast cancer cells (SUM-149-COX-2FL). Empty vector transduced SUM-149 cells (SUM-149-EV) were used for comparison. Higher basal and TPA-induced COX-2 mRNA and protein expression were confirmed in these cells (Supplementary Figures 1A and 1B). To evaluate the functionality of overexpressed COX-2 in the cells we measured secreted PGE_2_ levels and observed significantly higher basal and TPA-induced PGE_2_ secretion in these cells (Figure 7A). Tumors derived from these cells also expressed increased mRNA transcript and expressed higher COX-2 protein (Supplementary Figures 1C and 1D).

To further validate the role of COX-2 expression in modifying the ECM, SHG microscopy was performed on sections obtained from SUM-149-EV and SUM-149-COX-2FL cell derived tumors. Denser Col1 fibers were observed in SUM-149-COX-2FL tumor sections compared to SUM-149-EV tumor sections as shown in the representative Col1 fiber images in Figure 7B. Quantification of fiber volume and inter-fiber distance confirmed a significant increase of fiber volume and a significant decrease of inter-fiber distance with COX-2 overexpression (Figure 7C).

COX-2 overexpression in SUM-149 tumors increased the number of CAFs detected in the tumors as shown in the representative images obtained at 1X and 20X from SUM-149-EV and SUM-149-COX-2FL tumor sections stained for α-SMA (Figure 7D). This increase was confirmed following quantification of immunostained sections (Figure 7E). To further understand the role of COX-2 on changes in Col1 fiber content, human mammary fibroblasts (HMFs) were exposed to PGE_2_ and Col1A1 expression levels were determined. Exposure to PGE_2_ resulted in increased Col1A1 expression (Supplementary Figure 2).

COX-2 overexpression in SUM-149 cells did not significantly increase spontaneous metastasis in the axillary lymph nodes (data not shown). Both SUM-149-EV and SUM-149-COX-2FL cells formed emboli in the vasculature, and pulmonary metastasis. COX-2 overexpression did not increase the size of the emboli or pulmonary metastasis, following intravenous injection of the cells (Figures 7F and 7G). COX-2 overexpression did, however, significantly increase (p<0.05) the number of pulmonary metastatic nodules. Mean ± SEM values of the number of pulmonary metastatic nodules established by SUM-149-COX-2FL cells was 5.4±1.56, n=5, *versus* 2±0.83, n=5, established by SUM-149-EV cells.

## DISCUSSION

Here we have shown that downregulating COX-2 expression significantly impacted ECM structure, by reducing Col1 fiber volume, and ECM function, by altering permeability and macromolecular transport, in MDA-MB-231 tumors. Decrease of VEGF was identified as a mechanism by which vascular permeability decreased. COX-2 downregulation reduced the ability of these TNBC cells to form metastatic lung nodules and to metastasize to lymph nodes. Additionally, a significant decrease of Col1 fibers was observed in metastatic lung nodules established by COX-2 downregulated cells. To identify the cause of Col1 fiber reduction, we determined the number of CAFs in primary tumors and metastatic lung nodules. Consistent with the decrease of Col1 fibers, a significant reduction of CAFs was observed in COX-2 downregulated MDA-MB-231 tumors with a similar trend in metastatic nodules. A significant correlation was observed between the size of the nodule and the number of CAFs, identifying the importance of activated fibroblasts in the formation of metastasis, and the role of COX-2 in activating fibroblasts.

Downregulating COX-2 resulted in a significant delay of tumor onset of several days as well as slower growth. Although significant, the delay was not as profound as the delay of several weeks observed when COX-2 was completely silenced [17] further confirming the critical dependence of tumors on the prostaglandins produced by the enzyme for growth and progression [3].

Prostanoids produced by COX-2 such as PGE_2_ have been observed to mediate changes in angiogenesis [29–31], and anti-inflammatory agents have been found to have an antiangiogenic effect [31, 32]. Silencing of COX-2 in MDA-MB-231 cells downregulated several angiogenesis related transcripts [19]. COX-2-silenced MDA-MB-231 cells failed to promote the characteristic self-association patterns of endothelial cells in a co-culture model [19]. Here, a significant decrease of permeability and VEGF was observed in COX-2 downregulated MDA-MB-231 tumors. PGE_2_ is a major regulator of vascular permeability [31, 33], and the decrease of vascular permeability is consistent with the decrease of PGE_2_ and VEGF production observed in these tumors. Interestingly, macromolecular transport through the ECM was also significantly reduced with COX-2 downregulation, indicating functional modulation of the ECM by COX-2.

Previous studies have shown a marked alteration of the degradome and invasion associated transcripts, including a several fold down regulation of MMP1 [19], following COX-2 silencing in MDA-MB-231 cells. Here, COX-2 downregulated MDA-MB-231 cells showed a marked decrease in the ability to invade and colonize the lungs. Colonization of lymph nodes was also attenuated. These data further support the use of COX-2 inhibition to attenuate the metastatic cascade for those tumors that are COX-2 dependent.

We observed that the COX-2 downregulated MDA-MB-231 tumors had significantly sparser Col1 fiber distribution. These data further confirm earlier observations that COX-2 pharmacological inhibition reduces collagen deposition during mammary gland involution [22]. Col1 fiber density and orientation are increasingly being linked to breast cancer metastasis [25] and increased collagen content may contribute to the negative prognostic value of COX-2 expression in breast cancer patients [34, 35]. Col1 fibers were identified as channels that facilitate the ameboid movement of MDA-MB-231 cancer cells [36]. Reduced Col1 fibers have been previously associated with decreased macromolecular transport [37], and the reduced Col1 fibers in COX-2 downregulated tumors may have altered macromolecular transport in these tumors. Reduction of Col1 fibers was observed in metastatic nodules established by Clone 13 cancer cells following intravenous injection, suggesting that Col1 fibers are also important in the establishment of metastasis following extravasation.

Further confirmation of the role of COX-2 in modifying the ECM was evident from the significantly increased Col1 fiber density and volume observed in tumors derived from COX-2 overexpressing SUM-149 cells. COX-2 overexpression resulted in a significant increase of the number of pulmonary metastases, further supporting the role of COX-2 in establishing metastasis.

We used the expression of α-SMA to detect CAFs [38] in primary tumors and metastatic lung nodules. CAFs are a major source of Col1 fibers in the tumor stroma and contribute to the reactive desmoplastic tumor stroma [27]. CAFs play an active role in breast cancer metastasis [39, 40]. Here, for the first time, we observed that COX-2 downregulation in TNBC cells resulted in a significant decrease of CAFs in primary tumors derived from these cells, and in metastatic lung nodules. Conversely, COX-2 overexpression resulted in an increase of CAFs in primary SUM-149 tumors derived from these cells. The COX-2 dependent increase or decrease of CAF numbers may primarily explain the decrease in Col1 fiber content with COX-2 downregulation and the increase in Col1 fiber content with COX-2 overexpression. These data are consistent with a significant reduction of Col1 fibers observed following treatment of tumors with the antifibrotic agent Pirfenidone that eliminated CAFs [41]. PGE_2_ formed by COX-2 also increased Col1A1 expression in HMFs [42], although opposing effects have also been observed depending upon the type of fibroblast investigated [43].

Our studies were performed with TNBC cells, but future studies with ER/PR/HER2 positive breast cancer cells should further expand our understanding of the role of COX-2 in modifying the ECM and CAF numbers.

The role of CAFs in the establishment of metastasis was evident from the strong correlation between the size of the nodule and the number of CAFs present in the nodule. These results are also consistent with recent studies identifying the symbiosis between cancer cells and CAFs in tumor progression [44]. In addition to identifying the role of COX-2 in activating fibroblasts, our data suggest that including CAF immunostaining of breast cancer specimens may assist in identifying more aggressive cancers. The data also support disrupting cancer cell-fibroblast interactions as a strategy to arrest tumor growth and metastatic dissemination.

Collectively these data expand our insights into the role of COX-2 in breast cancer and its impact on the structure and function of the ECM. These insights are important as changes in the ECM and CAFs may occur during the course of treatments that upregulate COX-2 [45]. Our data identify a close dependence between COX-2 expression and the number of CAFs in primary tumors and metastatic nodules, and identify cancer-cell fibroblast signaling disruption as a potential treatment strategy to prevent metastatic dissemination.

## MATERIALS AND METHODS

### Stable expression of the COX-2 shRNA-containing plasmid in MDA-MB-231 cells

MDA-MB-231 breast cancer cells were obtained from ATCC (ATCC, Manassas, VA) and maintained in RPMI 1640 medium (Mediatech, Manassas, VA) supplemented with 10% fetal bovine serum (Sigma-Aldrich, St. Louis, MO). The COX-2 shRNA-coding plasmid was constructed and placed under the control of the U6 promoter as previously described [17]. Individual clones were selected for G418 resistance and analyzed for PGE_2_ production from the supernatant using the PGE_2_ enzyme immunoassay (EIA) Kit-Monoclonal as described by the manufacturer (Cayman Chemical, Ann Arbor, MI). Cells were induced for COX-2 expression by exposure to 50 nM TPA for twenty-four hours. Clone 13 cells were selected based on their significantly reduced basal and inducible COX-2 expression and PGE_2_ production.

### Overexpression of COX-2 in SUM-149 breast cancer cells

SUM-149 breast cancer cells were obtained from Asterand (Asterand, Inc., Detroit, MI) and maintained in Ham's F12 medium (SIGMA, St. Louis, MO) with 5% calf serum, insulin (5 μg/ml), and hydrocortisone (1 μg/ml). An ~1.8Kb region of the coding sequence of the human COX-2 gene (NM_000963.3) was PCR amplified and cloned into the PCR2.1 Topo vector (Invitrogen, Waltham, MA) and later subcloned between Xho1 and Kpn1 restriction sites in the multiple cloning site (MCS) of a pHAGE-pGK-MCS-Gtx-GFP lentivirus vector. 293T cells (ATCC, Manassas, VA) were co-transfected with the pHAGE-COX-2FL-Gtx-GFP plasmid, the ΔR8.2- packaging plasmid, and a plasmid expressing vesicular stomatitis virus glycoprotein (VSVG) to produce virions. Supernatant containing virions was added to SUM-149 breast cancer cells to derive cells stably expressing the COX-2 gene (SUM-149-COX-2FL). An empty vector without the gene was used to derive control cells (SUM-149-EV). Stable increase of COX-2 expression was verified by PCR and western blot analysis.

### Effect of PGE_2_ on Col1A1 expression in HMFs

HMFs kindly provided by Dr. Gary Luker, University of Michigan, Ann Arbor, were cultured in DMEM medium containing 10% fetal bovine serum (SIGMA, St. Louis, MO). For Col1A1 protein expression, HMFs were seeded in three 100mm dishes at 1.3×10^6^ cells per dish. Once the cells attached to the dish, cells were serum starved for twenty-four hours. At the end of serum starvation, PGE_2_ was added to two dishes at 3 ng/ml or 30 ng/ml in serum-free DMEM for an additional forty-eight hours. Untreated and PGE_2_ treated cells were analyzed for Col1A1 expression.

### Protein and mRNA expression

Expression levels of COX-2, α-SMA, Col1A1, and VEGF were determined by immunoblotting after blocking with 5% nonfat milk, with goat anti-COX-2 antibody (1:500, Cayman Chemical, Ann Arbor, Michigan), a monoclonal antibody against α-SMA (Clone 1A4, 1:1000), a rabbit polyclonal antibody against Col1A1 (ORIGENE, Rockville, MD), or an anti-VEGF polyclonal antibody (1:2000, Millipore Temecula, CA), and visualized with HRP (horseradish peroxidase)-conjugated secondary antibodies using the SuperSignal West Pico Chemiluminescent substrate kit (Thermo Scientific, Rockford, IL). Monoclonal anti-GAPDH antibody (1:50,000 dilution, Sigma-Aldrich) was used as loading control.

Total RNA was isolated from cells and tumor samples using the QIAshredder and RNeasy Mini kit (Qiagen, Valencia, CA). cDNA was prepared using the iScript cDNA synthesis kit (Bio-Rad). cDNA samples were diluted 1:10 and real-time PCR was performed using IQ SYBR Green supermix and gene specific primers in the iCycler real-time PCR detection system (Quanta Bioscience, Gaithersburg, MD). All primers were designed using Beacon designer software 7.8 (PREMIER Biosoft, Palo Alto, CA). The expression of target RNA relative to the housekeeping gene HPRT1 was calculated based on the threshold cycle (Ct) as R = 2^-Δ(ΔCt)^, where ΔC_t_= C_t_ of target - C_t_ of HPRT1.

### Tumor studies

Tumors derived from parent MDA-MB-231 and Clone 13 cells, with lower basal and inducible COX-2 expression levels and SUM-149 cells expressing an empty vector (SUM-149-EV) or overexpressing COX-2 (SUM-149-COX-2FL) were studied *in vivo*. Approximately 2-3 × 10^6^ cancer cells in 0.05 ml of Hanks balanced salt solution (HBSS) (Sigma-Aldrich, St. Louis, MO) were inoculated in the mammary fat pad of female severe combined immunodeficient (SCID) mice. Growth curves were obtained using cells inoculated in 0.05 ml of Matrigel solution (8.8 mg/ml) (Sigma-Aldrich). Orthotopic tumors were used to investigate the relationship between COX-2 expression, macromolecular transport using MRI and Col1 fiber density and volume using SHG microscopy. Lymph nodes excised from euthanized tumor-bearing mice were fixed in formalin, embedded in paraffin, sectioned at 5 μm thickness, and stained with H&E to evaluate for spontaneous metastasis. Separate sets of mice were injected intravenously with 10^6^ MDA-MB-231 or Clone 13 cells in 0.05 ml of HBSS. Tail vein injected mice were euthanized eight weeks later and metastatic burden and Col1 fibers in the metastatic nodules were evaluated from 0.5% agarose infused lungs that were fixed in formalin, embedded in paraffin, and sectioned.

All surgical procedures and animal handling were in accordance with protocols approved by the Johns Hopkins University Institutional Animal Care and Use Committee.

### MRI

Mice were imaged once tumor volumes were approximately 400-500 mm^3^. Mice were anesthetized, and a home-built catheter was inserted in the tail vein to inject the macromolecular contrast agent, albumin-gadolinium-diethylenetriaminepentaacetic acid (albumin-GdDTPA). MRI was performed on a 4.7 T Bruker spectrometer using a home built solenoid coil placed around the tumor. The respiration rate was monitored, and an isoflurane mask was used to maintain stable anesthesia during the 140 min of MRI scan time. The MRI acquisition was performed on volume-matched tumors as previously described [46]. Briefly, multi-slice relaxation rates (T_1_^-1^) were acquired using a saturation recovery technique with fast-T_1_ SNAPSHOT FLASH imaging (flip angle = 10 degrees, echo time = 2 ms). At the end of the MRI acquisition, blood T_1_ was determined from 20 microliters drawn from the tail vein. Images of the central 4 slices (slice thickness of 1 mm) of the tumor were acquired (128 × 128 matrix, 16 mm field of view, number of average = 8) for three relaxation delays (100, 500 and 1000 ms). A multislice map of completely relaxed magnetization (M_0_ map) was also acquired with a recovery time of seven seconds. The in-plane resolution of the MR images was 125 μm x 125 μm. Macromolecular transport parameters were measured from quantitative T_1_ maps obtained before and following intravenous administration of albumin-GdDTPA (500 mg/kg dose). Images were acquired in two “phases”. The “early phase” acquisition images included a pre-contrast image, and a 3-minute post-contrast image that was repeated every 5 minutes over the initial 30 minutes to characterize the tumor vascular volume and permeability surface area product. A second block of MR data, acquired up to 140 minutes post-contrast, was used to characterize the macromolecular transport parameters through the ECM. These transport parameters included the number of draining and pooling voxels, draining and pooling rates, and exudate volumes, derived as previously described [46]. A draining voxel was defined as a voxel in which the contrast agent accumulated at a rate lower than the permeability surface area product (PS), and a pooling voxel was one in which the contrast agent accumulated at a rate higher than the PS. After identifying the draining and pooling voxels, the influx and efflux rates were calculated. All quantification analysis was done in a home-built program written in IDL (ITT Exelis Visual Information Solutions, Herndon, VA) and AFNI (NIH software).

### Microscopy

Multiphoton microscopy was used to detect the SHG signal from Col1 fibers in 1 mm thick fresh tissue slices. SHG imaging was performed as previously described [47]. Briefly, we used a 25×/0.8 LD LCI PlanApo multi-immersion lens on a Zeiss 710 LSM NLO Meta multiphoton microscope (Carl Zeiss MicroImaging, Inc, Thornwood, NY). 3-dimensional (3D) image stacks were acquired from at least 10 randomly selected fields of view (FOVs) for each tumor. Following optical imaging, 5 μm thick adjacent sections were obtained from the optical slice and stained with H&E or with anti-α-SMA antibody as detailed in the immunostaining section.

Inflated lungs from mice in the experimental metastasis study were fixed with formalin. Five μm thick sections were obtained and stained with H&E or with anti-α-SMA antibody. Multiphoton microscopy was used to detect SHG signal from Col1 fibers in the H&E sections of the lungs from at least five randomly selected FOVs from each lung, using an Olympus Laser Scanning FV1000MPE multiphoton microscopy (Olympus Corp., Center Valley, PA) with a 25Xw/1.05XLPLN MP lens.

Col1 fiber distribution analysis was performed as previously described [47] by quantifying inter-fiber distance and percent fiber volume using a customized program written in Matlab (MATLAB 7.4.0, The MathWorks, Natick, MA).

Metastatic burden was calculated as [(Total area of metastatic foci in μm^2^)/(Total lung area in μm^2^)]x100 from high-resolution digital scans of the H&E sections obtained using ScanScope (Aperio, Vista, CA). Images were processed with ImageScope software (Aperio). Col1 fiber distribution in these sections was performed as described earlier using SHG microscopy data acquired from size-matched metastatic lung nodules to avoid nodule size bias.

### Fibroblast immunostaining

Immunohistochemistry of tumor sections was performed using the streptavidin–peroxidase technique and the DAKO EnVision System (Dako Cytomation, Hamburg, Germany) as previously described [48] using the alkaline conjugated monoclonal anti-α-SMA antibody (clone 1A4) primary antibody (Sigma; 1:200 dilution, 4°C overnight).

High-resolution digital scans of the immunostained sections were obtained using ScanScope (Aperio). Images were processed and nuclei and membrane intensity quantified with ImageScope software using the algorithm supplied by the manufacturer (Aperio). The number of CAFs was quantified by computing the fraction of intensely stained pixels in the FOVs. Intensities from vessel regions were excluded for quantification.

### Statistical analysis

Statistical significance was determined using an unpaired Students t-test performed using Microsoft Office Excel 2010 (Microsoft, Redmond, WA). P values ≤ 0.05 were considered significant unless otherwise stated. To determine if the total metastatic nodule area was associated with strong α-SMA expression, *i.e*. the number of activated fibroblasts, we computed the Spearman rank correlation coefficient between these data for both tumor types.

## SUPPLEMENTARY MATERIALS FIGURES



## Abbreviations

Albumin-GdDTPA: albumin-gadolinium-diethylene-triaminepentaacetic acid
CAFs: cancer associated fibroblasts
Col1: collagen 1
COX-2: cyclooxygenase-2
ECM: extracellular matrix
EIA: enzyme immunoassay
FOV: field of view
GAPDH: glyceraldehyde 3-phosphate dehydrogenase
H&E: hematoxylin and eosin
HMFs: human mammary fibroblasts
HRP: horseradish peroxidase
kDA: kilodalton
MRI: magnetic resonance imaging
PBS: phosphate buffered saline
PGE_2_: prostaglandin E2
PS: permeability surface area product
SCID: severe combined immunodeficient
SDS-Page: sodium dodecyl sulfate poly-acrylamide gel electrophoresis
SHG: second harmonic generation
shRNA: short hairpin RNA
siRNA: small interfering RNA
SMA: smooth muscle actin
TNBC: triple negative breast cancer
TPA: 12-O-tetradecanoylphorbol-13-acetate
VEGF: vascular endothelial growth factor
