# Supplementary material for: Breast cancer cell cyclooxygenase-2 expression alters extracellular matrix structure and function and numbers of cancer associated fibroblasts
Source: Oncotarget. 2017 Jan 31;8(11):17981–94. doi: 10.18632/oncotarget.14912 (PMC5392301; doi:10.18632/oncotarget.14912)
Supplement: Supplementary file 1 [file oncotarget-08-17981-s001.pdf]

Breast cancer cell cyclooxygenase-2 expression alters extracellular matrix structure and function and numbers of cancer associated fibroblasts

SUPPLEMENTARY MATERIALS

Cloning, generation of virus and transduction of SUM149 expressing COX-2

The full length fragment of human cyclooxygenase 2 (NM_000963.3) measuring about 1.8kb was cloned into a lentiviral vector pHAGE-Gtx-GFP between the Xho1 and Kpn1 sites, situated 5'to the internal ribosome entry site Gtx. The production of viral supernatant, and the transduction method used here have been previously described (Krishnamachary *et al.,* Cancer research 2009; 69: 3464-71). Viral supernatant was added to SUM 149 breast cancer cells (Asterand, Inc., Detroit, MI), and transduction was achieved in the presence of polybrene (8µm/ml). Forty-eight hours post transduction, cells were evaluated for GFP expression. Genetically modified SUM-149 cells were characterized for expression of COX-2 protein and PGE_2 activity. A viral construct lacking the insert was used as an empty vector control. All cell lines used in this study were authenticated by the short tandem repeat (STR) profile kit (GenePrint 10, Promega) at the genetic resource core facility of Johns Hopkins University, Baltimore, USA.

Primers used in this study

All primer sequences were designed using Primer 3Plus software.

(1) Human COX-2 (NM_000963)

SYBR green based QuantiTect primer was purchased from Qiagen- (Cat. No- QT00040586, Valencia, CA)

(2) Human HPRT1 (NM_000194.2)

Human HPRT1 Forward- 5'- CCTGGCGTCGTGATTAGTGATG -3'

Human HPRT1 Reverse-5'- CAGAGGGCTACAATGTGATGGC -3'

SUPPLEMENTARY FIGURES

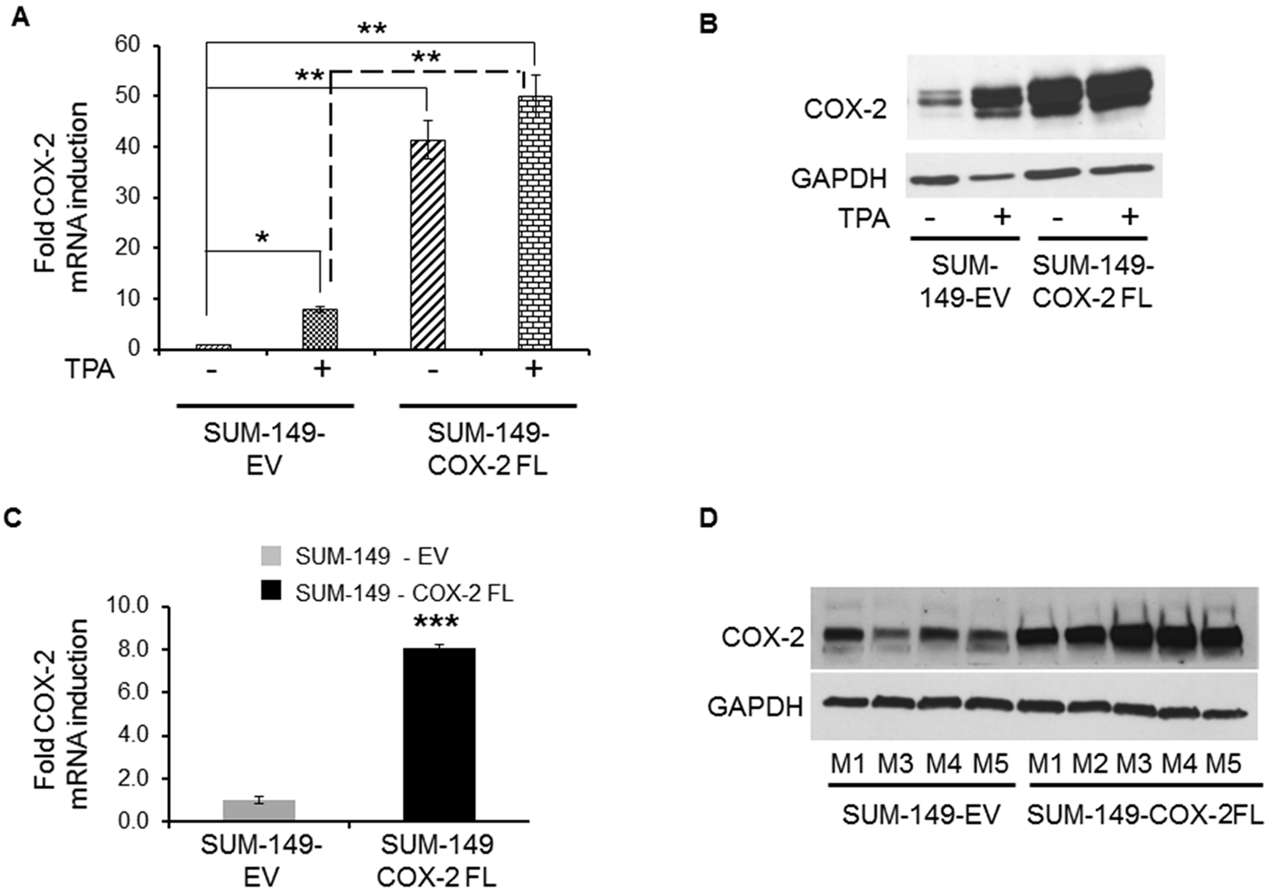

Supplementary Figure 1: A. Relative fold changes in COX-2 mRNA transcript levels in response to TPA treatment in SUM-149-EV cells and in COX-2 overexpressing SUM-149-COX-2-FL cells. Values represent Mean ± SEM from three independent experiments. * p≤ 0.05, ** p≤ 0.005 using ΔC_t values. **B.** Representative immunoblot showing increase of COX-2 expression in SUM-149 breast cancer cells stably over expressing COX-2. GAPDH was used as a loading control. **C.** Relative fold change in COX-2 mRNA levels in SUM-149-EV (n=5) and SUM-149-COX-2FL (n=5) tumors. Values represent Mean ± SEM. * p≤ 0.05; *** p≤ 0.0005 using ΔC_t values. **D.** Representative immunoblot showing COX-2 expression in SUM-149-EV and SUM-149-COX-2FL tumors. GAPDH was used as a loading control.

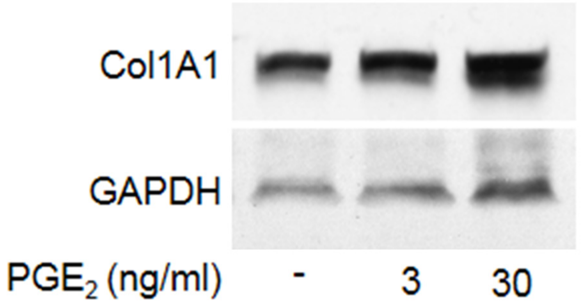

Supplementary Figure 2: Representative immunoblot showing increase in Col1A1 protein expression in HMFs in response to 3 ng/ml and 30 ng/ml of PGE_2 treatment for 48h following serum starvation, compared to control cells maintained under serum free condition. GAPDH was used as a loading control.
